# High energy tibial plateau fractures treated with hybrid external fixation

**DOI:** 10.1186/1749-799X-6-35

**Published:** 2011-07-14

**Authors:** George C Babis, Dimitrios S Evangelopoulos, Panagiotis Kontovazenitis, Konstantinos Nikolopoulos, Panagiotis N Soucacos

**Affiliations:** 1 A' Orthopaedic Department University of Athens, Attikon University Hospital, Athens, Greece; 2 C' Orthopaedic Department, University of Athens, KAT Accidents' Hospital, Athens, Greece; 3 Associate Professor, C' Orthopaedic Department, University of Athens, KAT Accidents' Hospital, Athens, Greece

## Abstract

Management of high energy intra-articular fractures of the proximal tibia, associated with marked soft-tissue trauma, can be challenging, requiring the combination of accurate reduction and minimal invasive techniques. The purpose of this study was to evaluate whether minimal intervention and hybrid external fixation of such fractures using the Orthofix system provide an acceptable treatment outcome with less complications. Between 2002 and 2006, 33 patients with a median ISS of 14.3 were admitted to our hospital, a level I trauma centre, with a bicondylar tibial plateau fracture. Five of them sustained an open fracture. All patients were treated with a hybrid external fixator. In 19 of them, minimal open reduction and stabilization, by means of cannulated screws, was performed. Mean follow-up was 27 months (range 24 to 36 months). Radiographic evidence of union was observed at 3.4 months (range 3 to 7 months). Time for union was different in patients with closed and grade I open fractures compared to patients with grade II and III open fractures. One non-union (septic) was observed (3.0%), requiring revision surgery. Pin track infection was observed in 3 patients (9.1%).

Compared to previously reported series of conventional open reduction and internal fixation, hybrid external fixation with or without open reduction and minimal internal fixation with the Orthofix system, was associated with satisfactory clinical and radiographic results and limited complications.

## Introduction

Intra-articular fractures of the proximal end of the tibia, the so-called 'plateau fractures', are serious, complex injuries difficult to treat [1]. The mechanism of injury is based on the presence of an initial axial load, which fractures the tibial articular surface resulting in impaction. In most of the cases the initial load is combined with angular forces, leading to comminution not only of the articular surface, but of the metaphysis as well. The medial compartment is split in a medio-lateral direction with a postero-medial main fragment, combined with various amounts of multifragmental lateral compartment depression [2].

According to Schatzker's classification [3,4], these fractures are divided into six groups: S-I to S-VI. Of these types, those involving both condyles (S-V) and those separating tibial metaphysis from diaphysis (S-VI) are the most challenging fractures for the Orthopaedic Surgeon to treat not only for the osseous damage but for the restoration of the soft tissue envelope as well.

Standard radiographic imaging includes anteroposterior and lateral views. Suspicion of distal extension of the fracture mandates that full-length tibia and fibula x-rays should be obtained. The CT scan is becoming more and more useful in the evaluation of the size, comminution and orientation of the articular fragments, allowing proper classification and preoperative planning, thus facilitating reduction, especially for the less invasive techniques of treatment [5].

Over the years, many treatment modalities have been proposed for these complex fractures. All of them, from simple traction to demanding surgery, presented fair results but also serious complications.

Traction, in terms of ligamentotaxis and casting, do not properly reduce the articular surface and lack the necessary stability, leading to unacceptable rate of varus/valgus deformity, collapsed articular surface and post-immobilization stiffness [6-9]. On the other hand, open surgical procedures, despite their good reduction results, do not protect the already damaged "soft-tissue envelope", leading to skin or muscle necrosis and to high rates of infection [10,11].

The use of a "minimal invasive technique", an external fixator, in the treatment of S-V and S-VI fractures may provide fair reduction results without endangering the soft-tissue elements. Moreover, it facilitates the access to any endangered soft tissue elements requiring interventions along the treatment period. The addition of minimal internal fixation with cannulated screws and k-wires prior to an external fixator application provides minimum soft tissue striping and greater fixation stability, allowing for early mobilization and greater range of motion [12-17].

The purpose of the current study was to test the hypothesis whether minimal intervention and hybrid external fixation using the Orthofix system can provide a fair outcome with less complications and to compare our results and complications with previously reported data of internal and external fixation for types V and VI high energy tibial plateau fractures.

## Materials and methods

After receiving approval from our Institutional Review Board, we retrospectively examined a consecutive series of 33 patients (33 bicondylar tibial plateau fractures (Schatzker type V, VI) admitted at our level I trauma centre between 2002 and 2006. Fractures were identified through our trauma database and were cross-matched with operating room records. Median ISS was 14.3, ranging from 9 to 33. Inclusion criteria were the presence of a bicondylar tibial plateau fracture Schatzker type V-VI, patients' age over 18 years and the ability to walk without assistance before injury. Polytrauma patients with tibial plateau fractures requiring prolonged ICU care (AIS>3 for head and chest) and patients with bilateral plateau fractures, were excluded from the study. All patients were followed according to a protocol. All fractures were treated with either closed reduction and hybrid external fixation (14 fxs/36.6%) or with minimal open reduction and a hybrid system (19 fxs/63.4%). The study group was consisted of 20 males (60.6%) and 13 females (39.4%) with an average age for males of 40.3 years (range 30 - 62 years) and for females 49 years (range 17 - 86 years). In 27 patients (81.8%) the mechanism of injury was high energy trauma (motor vehicle accident or fall from height greater than 3 m). All patients had anteroposterior and lateral radiographs as well as a CT-scan for proper preoperative evaluation of their fracture.

The preoperative radiographs were used to classify the fractures according to Schatzker's classification system. There were 16 S-V (48.5%), and 17 S-VI (51.5%) fractures. Twenty eight (28) were closed (84.8%) and five (5) were open fractures (15.2%). Of those, one (1) fracture was type I, two (2) type II and two (2) were type IIIA open fractures according to Gustilo-Anderson classification. Peroneal nerve injury occurred in one (1) patient (3.0%), at the time of the injury. Two patients (6.0%) had major knee instability with rupture of ACL and LCL.

Nineteen (19) patients (57.5%) were submitted to minimal open reduction by means of cannulated screws prior to the application of an external fixator. In seventeen (17) of these patients (51.5%), cortical allografts were used. All patients were available for follow up (average 27 months, range 24 - 36 months) with repeated anteroposterior and lateral radiographs at 1.5, 3, 6, 12, 18, 24 and 36months.

Soft tissue condition had a crucial importance on our planning for the time of the operation. All patients with open fractures (5) (15.2%) were operated immediately with irrigation, debridement, intravenous antibiotics. 18 (54.5%) closed fractures were treated in the first day after the accident while 7 fractures (21.2%) were treated with an average of 5 days delay (range 3 - 9 days) in order to allow soft tissue edema to subside. For the latter group a posterior long leg splint was placed to the affected limb.

Prophylactic antibiotics were administered intravenously in all cases. In the open fracture cases, antibiotics were prescribed as necessary for the first days and subsequently replaced according to the cultures results. All open fractures received initially a combination of a 2^nd ^generation cephalosporin with an aminoglycoside. Both open and closed fractures received preoperatively a single dose of teicoplanin.

## Surgical technique

**Figure 1:**
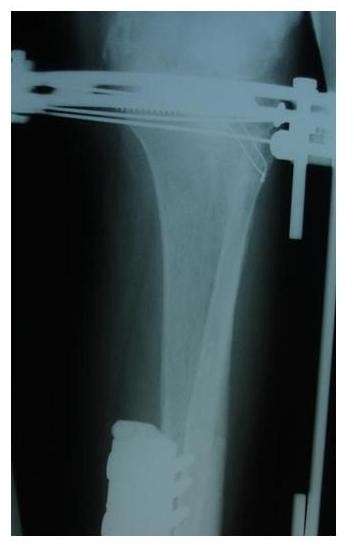
**Postoperative AP x-ray of a male patient demonstrating a Schatzker's VI tibial plateau fracture of the left lower limb**.

**Figure 2:**
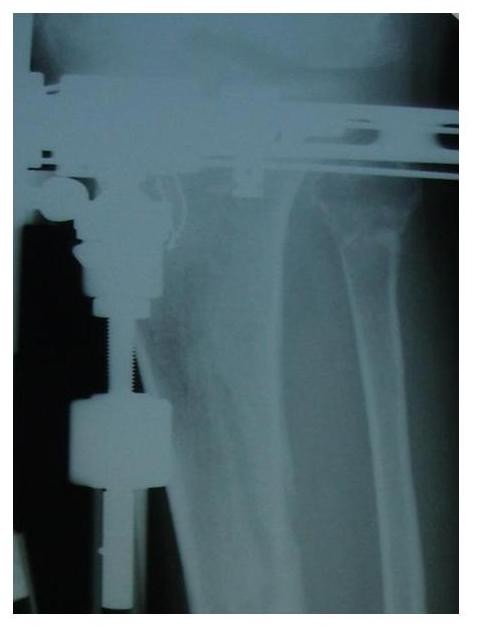
**Postoperative lateral x-ray of the same patient**.

We used the Orhtofix hybrid external fixation system. Surgery was performed under general or spinal anesthesia with the patient positioned on the operating table with the knee flexed at 30°. A tourniquet is not a significant advantage in closed reduction, but if used, should be deflated as soon as possible. The fracture reduction was visualized with an image intensifier. Through a small incision over the antero-medial aspect of the tibial metaphysis, a small "window" was made in the tibial cortex. A blunt tipped curved 3 mm k-wire or a simple pusher was inserted through the hole, up to the articular fragments, which were elevated under image intensifier control. In most of the cases, more than one k-wire was required to reduce the articular fracture. Bone grafts were applied to feel osseous gaps. Through a small lateral incision, a Kirschner wire was inserted across the tibial plateau to stabilize the reduced fragments and a cannulated screw was introduced over it. After closed or minimal open reduction of fracture fragments, an Orthofix hybrid external fixator was applied. A ring of appropriate size was positioned at the level of the fibular head. All wires were applied in the transverse plane, 2 from lateral to medial and the remaining 2 from antero-lateral to postero-medial. Each wire was tensioned to 1,400 N and locked to the frame. The metaphyseal fracture was reduced accurately and the body of the external fixator was applied on the ring on the antero-medial aspect of the tibia. Two pin guides were inserted down to the skin which was then incised. Pin holes were pre-drilled with a 4.8 mm drill bit and three 5/6 mm tapered self-tapping cortical pins were inserted. The fixator was clamped to the screws. It was of crucial importance that the fracture was reduced before the permanent fixation of the hybrid system. After achieving adequate reduction, the system was locked and secured. The reduction was then confirmed by C-arm. If alignment was not satisfactory, a minimal exposure of the fracture site was performed to enable the desirable reduction (Figure 1, 2).

For mini open exposures, wound was closed primarily for close fractures. For open fractures, we preferred either to leave the wound open for surgical debridement or to proceed to a delayed primary closure 72 h postoperatively. Skin graft coverage was needed only for one patient (S-V(G-IIIA).

Post-operative care consisted of daily performed thorough pin care, from the first postoperative day, with hydrogen peroxide and betadine as well as immediate passive range of motion of the knee. For highly comminuted fractures, a posterior splint was applied and after 48 hours the patient was encouraged to start controlled knee movement as soon as possible. Patients were discharged from the hospital between the 5th and 15th postoperative day, depending on their general condition. Patients with Gustilo grade II and III open fractures were checked weekly in the outpatient department. All the other patients were checked monthly. They were instructed not to bear weight on the operated limb and to regularly perform pin site care. Progressive weight bearing was allowed between the 8th and 12th week depending on the radiographic appearance of callus. The weight bearing started with 10 kg and, based on the clinical and radiographic signs of union, advanced to 30 kg after one month. In most of our cases, the external fixator was removed at 3.4 months after surgery depending on the radiological appearance of union.

## Results

Patient results are given in Table 1. All fractures in this series except one (3.0%) healed. Union was determined by the presence of a bridging callus on the follow-up radiographs and by the clinical impression of stability. Follow-up evaluation was available for all fractures. Based on the parameters considered at the follow-up (radiological results, knee ROM, pain, ability to perform sport activities, and patient's satisfaction), according to KSS criteria [18], the results were evaluated as excellent in 18 patients (55%), good in 10 patients (30%), fair in 4 patient (12%), and poor in 1 (3%) (Table 1).

**Table 1:** Fractures' characteristics, complications and results of our study group.

No of pts	Schatzker type	Open/ closed	Results	Complications
1	V	closed	excellent	None

2	V	closed	excellent	None

3	V	closed	good	pin track infection-per os antibiotics

4	V	closed	excellent	None

5	V	closed	excellent	None

6	V	open: G-I	excellent	None

7	V	closed	excellent	None

8	V	closed	good	None

9	V	closed	excellent	deep venous thrombosis

10	V	closed	excellent	None

11	V	closed	good	None

12	V	closed	excellent	None

13	V	closed	excellent	None

14	V	closed	excellent	None

15	V	closed	excellent	None

16	V	closed	excellent	None

17	VI	closed	good	None

18	VI	closed	excellent	pin track infection- per os antibiotics

19	VI	open:G-III	fair	local skin necrosis

20	VI	closed	excellent	None

21	VI	open: G-II	good	pin track infection- per os antibiotics

22	VI	closed	good	None

23	VI	closed	good	None

24	VI	open:G-II	fair	malunion, 10° valgus

25	VI	closed	excellent	None

26	VI	open:G-III	fair	traumatic peroneal nerve palsy

27	VI	closed	good	None

28	VI	closed	fair	malunion, 5° procurvatum

29	VI	closed	excellent	None

30	VI	closed	excellent	None

31	VI	open: G-III	poor	deep infection- septic pseudarthrosis

32	VI	closed	good	None

33	VI	closed	good	None

**Figure 3:**
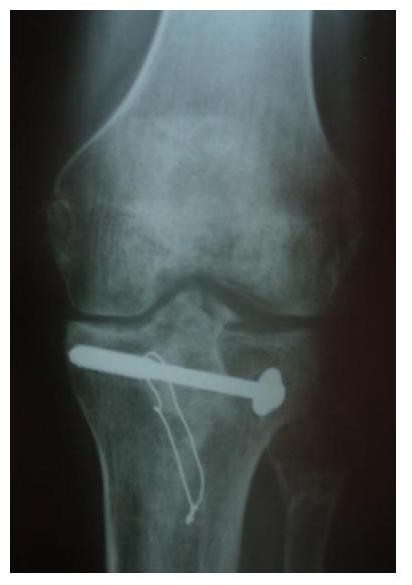
**AP x-ray of the same patient after hybrid external fixator removal**.

**Figure 4:**
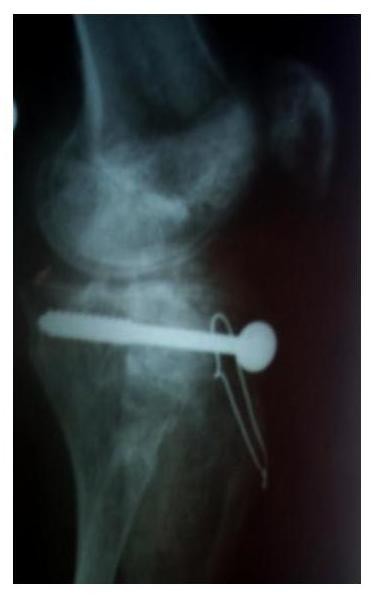
**Lateral x-ray of the same patient after hybrid external fixator removal**.

There were no systemic complications attributable to our surgical treatments. All associated ligamentous and meniscal lesions were repaired at a second stage after fracture healing. All fractures healed, with an average time of treatment with the frame of 3.4 months (Figure 3, 4). The external fixator was tolerated for the entire treatment period in all cases. Two fractures (6.0%) took longer than 6 months to heal.

In our series only one (1) fracture was complicated with deep infection leading to septic non-union (3.0%). It was treated with surgical debridement and i.v. antibiotics until CRP and ESR reached normal values. Later on, open reduction and internal fixation with plate and autologus bone grafting was performed. Deep venous thrombosis was detected in one patient (3.0%) and was treated successfully with low molecular weight heparin.

There were 3 pin track infections (9.1%). These infections were superficial or limited to the soft tissue and did not extend to the bone. None of the patients required hospital admission. There were treated with oral antibiotic and local pin care. All pin track infections healed without requiring wire or half-pin removal that could compromise frame's stability. Two fractures (6.0%) resulted in malunion (10° of valgus, < 5° procurvatum), but faced no symptoms. In one case of an open fracture, local skin necrosis occurred requiring a skin graft.

A total of 26 (78.8%) patients regained functional use of the knee joint, good axis, without pain or instability. Patients' knee ROM was gradually increasing at consecutive clinical evaluations. Patients were discharged from the hybrid fixator after an average time of 3.4 months (range 3 - 7 months). At the one year follow-up, range of motion averaged 115° of flexion (range 75° to 125°) and 5° lack of extension (range 0°- 8°). During the radiographic follow-up evaluation, early osteoarthritic changes at the knee joint were noticed in one (1) patient (3.0%) (SVI/GII fracture).

Overall, 5 patients (15.1%) faced with at least 1 minor complication such as pin track infection, stiffness, malunion and 1 patient (3.0%) came up with at least one (1) major complication including septic-nonunion and osteomyelitis. No amputation was performed.

## Discussion

The importance of the soft-tissue envelope in the healing of plateau fractures has been analyzed in the literature and a correlation of poor results with severely damaged soft-tissues has already been established [19]. High energy trauma is considered as a major cause of poor results in the treatment of tibial plateau fractures. Different methods for treating these complex injuries have been proposed, including limited open reduction and stabilization with percutaneous screws, open reduction and internal fixation [4,20-23] and indirect reduction and application of a hybrid [24-26] or a circular external fixation device [27,28].

Internal fixation, despite the advantages of direct visualization, proper and stable reduction of the articular surface as well as the acute repair of soft tissue injuries, presents also serious disadvantages, including skin or soft-tissue necrosis caused by surgical manipulations on an already damaged soft-tissue envelope and the high rate of infection, which may compromise the final result. Tscherne et al, comparing the results of surgical versus conservative treatment for tibial plateau fractures, reported improved range of motion, decreased percentage of malunion and 5% reoperation rate for the surgical group [29]. Stevens et al, presented several transoperative - postoperative complications [30], while Young and Barrack, in their series of dual plating for complex bicondylar tibial plateau fractures reported an 88% deep infection rate [31,32]. Certain authors have treated bicondylar tibial fractures by means of a lateral fixed angular plate (FAP) through a single lateral approach, thus avoiding medial periosteal striping [33,34]. Jiang R et al, in their prospective study comparing locked plates, to classic double plates (DP), for the repair of bicondylar tibial plateau fractures reported similar results for the two groups [35]. Nevertheless, as presented by Higgins et al., bicondylar fractures stabilized by means of a FAP present a higher rate of subsidence compared to dual plating stabilized fractures [36].

The external fixation as a definite treatment for the polytrauma patient with multiple osseous and soft tissue injuries has been described in the literature [37,38]. Certain authors believe that external fixation should be limited to bicondylar tibial fractures with a compromised soft-tissue envelope, as a temporary stabilizing technique, prior to definite treatment [39]. In the last 2 decades, the evolution of devices and techniques of external fixation has led many surgeons to apply the principles of biologic osteosynthesis and minimally invasive surgery for the treatment of comminuted tibial plateau fractures [4,28,32,39]. The development of circular and hybrid frames, the capability of axial, lateral compression and dynamization, the development of olive wires have offered new possibilities to the external fixators for the treatment of complex fractures [40]. Mahadena et al, comparing external to internal fixation, concluded that hybrid external fixation possesses theoretical advantages in terms of the soft tissues protection; however the benefit over internal fixation is modest as far as accuracy of reduction is concerned [41]. Chin et al presented 38.9% good/excellent, and 61.1% fair/poor results in his type V and VI fracture series [42]. Catagni et al, in their series of high-energy Schatzker V and VI tibial plateau fractures treated with circular external fixator, reported excellent and good results in 30 (50.85%) and 27 (45.76%) patients respectively [23]. In a similar study on type V and VI tibial plateau fractures, Katsenis et al recorded excellent or good final clinical results in 36 patients (76%) [24]. In 2009, the Canadian Orthopaedic Trauma Association, in a multicenter, prospective, randomized clinical trial of 83 S-V, VI tibial plateau fractures treated with internal or external fixation, reported similar quality of osseous reduction and ROM for both groups but lower rate of early postoperative complications and improved HSS scores for the external fixation group at the six months' follow up. However, at the two years' follow up, no significant difference in ROM, HSS scores, WOMAC and SF-36 was observed between the two groups [43].

In our series, we used the Orthofix hybrid external fixator as a definite treatment for Schatzker V, VI closed fractures as well as for some open tibial plateau fractures. When necessary, open reduction and minimal internal fixation by means of k-wires or screws were performed prior to external fixation application. Overall, we had an incidence of infection of 12.1%. This rate compares favorably with historical controls as seen in table 1. The rate drops to 3.0% (1 pt) if we look only at deep infections. All the other cases (3 pts), were superficial pin tract infections that resolved with proper care and oral antibiotics. Malunion (valgus-procurvatum) was observed in two patients. It is important to note that the case of deep infection as well as the two cases of malunion occurred in the group of Schatzker VI-open fractures. In many older articles, authors do not break down their complications according to the type of the tibial plateau fracture [4,22,44]. Our cohort by contrast is essentially a homogeneous group composed of Schatzker V and VI fractures secondary to a high-energy mechanism. A similar homogenous group was presented by Covall et al. The authors treated 32 bicondylar tibial plateau fractures during a 7-year period and reported a 42% deep infection rate in the cases treated acutely with internal fixation [15].

As far as minor complications are concerned, Hutson et al, in a meta-analysis of 16 studies with a total of 568 patients found pin site infection rates of 10% for tibial plateau fractures [45]. This number is similar to the rate of pin tract infection (9.1%) observed in our series. Moreover, the two cases of malunion (6.1%) represent an acceptable rate as compared with other series [23]. Complications concerning the external fixation device such as intolerance or pin loosening were not observed in our study.

## Limitations

As limitations of this study, one should consider its retrospective nature. Additionally, since our study group is composed of high energy plateau fractures with a high complication rate, the average follow up of 27 months can be considered as inadequate to draw safe conclusions for the development of post-traumatic osteoarthritis. This report may be the basis for a new study examining the development of post- traumatic arthritis in patients with high energy plateau fractures.

## Conclusions

Schatzker's type V and VI tibial plateau fractures represent serious injuries with substantial residual limb-specific and general health deficits [43]. We believe that the use of Orthofix external fixation, as a definite treatment, for high-energy proximal tibia bicondylar fractures proved to be beneficial. While confronting such limb-threatening injuries, external fixation successfully provided continuous access on the surrounding tissues as well as proper osseous stabilization without compromising the sensitive soft tissue envelope.

## Competing interests

The authors declare that they have no competing interests.

## Authors' contributions

All authors contributed equally to this work. BGC, DSE, PK, KN and PNS participated in the design of the study and drafted the manuscript. BGC and DSE participated in the design of the study. BGC and KN conceived of the study, participated in its design and coordination and helped to draft the manuscript. All authors read and approved the final manuscript.
